# Long-Term Outcomes of Tension-Free Vaginal Tape Obturator: Efficacy and Safety at Long-Term Follow-Up

**DOI:** 10.3390/jcm13195699

**Published:** 2024-09-25

**Authors:** Andrea Braga, Andrea Papadia, Elena Gamarra, Giorgio Caccia, Maria Rosaria Campitiello, Marco Torella, Giada Mesiano, Martina Fiorani, Chiara Scancarello, Chiara Cimmino, Maurizio Serati

**Affiliations:** 1 Department of Obstetrics and Gynecology, EOC-Beata Vergine Hospital, 6850 Mendrisio, Switzerland; andrea.braga@eoc.ch (A.B.); giorgio.caccia@eoc.ch (G.C.); 2 Faculty of Biomedical Sciences, Università della Svizzera Italiana, 6900 Lugano, Switzerland; andrea.papadia@eoc.ch; 3 Department of Obstetrics and Gynecology, EOC–Civico Hospital, 6900 Lugano, Switzerland; 4 Department of Endocrinology and Diabetology, EOC–Civico Hospital, 6900 Lugano, Switzerland; elena.gamarra@eoc.ch; 5 Department of Obstetrics and Gynecology and Physiopathology of Human Reproduction, ASL Salerno, 84124 Salerno, Italy; rosycampitiello@gmail.com; 6 Department of Gyanecology, Obstetric and Reproductive Science, Second University of Naples, 80131 Naples, Italy; marcotorella@iol.it; 7 Department of Obstetrics and Gynecology, Del Ponte Hospital, University of Insubria, 21100 Varese, Italy; giadamesiano01@gmail.com (G.M.); martina.fiorani@gmail.com (M.F.); chiara.scancarello@asst-settelaghi.it (C.S.); chia.cimmino@gmail.com (C.C.)

**Keywords:** tension-free vaginal tape obturator, TVT-O, stress urinary incontinence, long-term follow-up, mid-urethral sling, tape exposure

## Abstract

**Background/Objectives:** The use of tension-free vaginal tape obturator (TVT-O) for the treatment of stress urinary incontinence (SUI) has been widely debated over the last decade due to the lack of evidence on its long-term outcomes. The aim of this prospective study is to assess, for the first time in the available literature, the efficacy and safety of TVT-O implantation in women with pure SUI over a 17-year follow-up period. **Methods**: We included all women who complained of pure SUI symptoms (confirmed urodynamically) and underwent the TVT-O procedure. An objective cure was defined as the absence of urine leakage during the stress test, while subjective outcomes were assessed by means of the International Consultation on Incontinence Questionnaire—Short Form (ICIQ-SF), the Patient Global Impression of Improvement (PGI-I) scale, and a Visual Analogue Scale (VAS). **Results:** A total of 70 patients who met the inclusion criteria underwent the TVT-O procedure. During the study period, no patients were lost to follow-up, and all women completed the last evaluation at the 17-year mark. At the 17-year mark of follow-up, 62 out of 70 patients (81.4%) were subjectively cured, and 56 out of 70 (80%) patients were objectively cured. These data do not reveal any significant variation in the surgical outcomes over the follow-up period. We recorded seven (10%) tape exposure (three occurred after 10 years and four after 17 years). Among these, one woman was symptomatic for dyspareunia and “hispareunia”. All patients with mesh exposure were treated with partial removal and re-suture of the vagina, but only one developed the recurrence of SUI that required a second treatment with a urethral bulking agent (UBA). In all other cases, women reported a complete resolution of symptoms without any worsening of the urinary continence. No significant bladder or urethral erosion was recorded. **Conclusions**: The 17-year evaluation of the TVT-O procedure has shown that it is a highly effective and safe option for the treatment of female SUI. Although there was an increased risk of tape exposure 17 years after implantation, no serious complications were reported, and no patient required the total removal of the sling.

## 1. Introduction

SUI is the most prevalent urinary incontinence type, ranging from 29% to 73%, with a mean of 48%. The burden of SUI is high in both human and economic aspects and has a negative impact on quality of life (QoL). It also contributes to depression, falls, and hospitalization [1].

Although the use of mid-urethral slings (MUSs) for the treatment of SUI has been widely debated over the last decade, this procedure continues to have a central role in the management of this global problem. In fact, as stated in the recommendations of the Urogynecology and Pelvic Floor Committee of the International Federation of Gynecology and Obstetrics (FIGO) [2], MUSs are considered safe and effective procedures, based on many high-quality scientific publications and leading professional society guidelines.

Nevertheless, there is a lack of evidence on the long-term outcomes of MUS to be able to recommend these procedures with a strong level of evidence. Furthermore, concerns about the use of the transvaginal mesh for pelvic organ prolapse (POP) also involved the slings intended for the treatment of SUI, especially regarding the TVT-O procedure. Astonishingly, the NICE guidelines [3] excluded this type of MUS among the first-line treatments of SUI, considering it less safe than the other options, mainly in terms of tape exposure. Nevertheless, several studies in the literature reported a low rate of long-term complications for this mesh. In a retrospective population-based cohort study, Gurol-Urganci et al. [4] included 95 057 women who underwent their first MUS insertions for SUI and reported a 9-year removal risk after transobturator insertion of 2.7%, which was lower than the risk after retropubic insertion (3.6%). Similarly, in a multicenter prospective study, we found a sling exposure rate of 2.5% at the 10-year follow-up [5]. Another recent analysis of patients included in the French multicenter register VIGI-MESH [6], for the prospective monitoring of serious complications of SUI and POP mesh procedures, showed a cumulative 2-year estimate of serious complications in 5.8% of patients who underwent retropubic MUS compared to 2.9% in the transobturator group. Although these data seem promising, long-term benefits need to be evaluated.

The aim of this prospective study is to assess, for the first time in the available literature, the efficacy of TVT-O implantation in women complaining of pure SUI at the 17-year follow-up. In addition, we evaluated the safety of this procedure classifying long-term complications according to the Clavien–Dindo system.

## 2. Materials and Methods

This is an observational prospective study performed in two tertiary reference centers. We included all women who complained of pure SUI symptoms (confirmed urodynamically) from January 2004 to December 2005 and underwent the inside-out TVT-O procedure (Gynecare TVT Obturator System; Ethicon Inc., Somerville, NJ, USA).

As previously described in similar evaluations [5,7,8,9], we excluded women with concomitant POP greater than stage 1 according to the POP quantification system [6], overactive bladder (OAB) symptoms, or destrusor overactivity (DO), excluding women with dysfunction (with postvoid residual > 100 ml) and a history of radical pelvic surgery or psychiatric and neurologic diseases.

During preoperative examination, we collected clinical history, urinalysis, and a 3-day bladder diary and performed a physical examination (including POP-Q system evaluation) and a complete urodynamic study (UDS) according to the Good Urodynamic Practice guidelines of the International Continence Society [10]. The UDS, performed by an experienced urogynecologist, comprised the following: uroflowmetry, filling cystometry, Valsalva leak-point pressure (VLPP) measurement, and pressure/flow study. Urethral hypermobility was referred to as Q-tip test > 30°. Women were included independent of their Q-tip and VLPP test values. The ICIQ-SF [11] was filled out by all patients at baseline and during follow-up. Surgical procedures were performed under general or spinal anesthesia, depending on anesthesiological requirements and/or patient preference [12]. Local estrogen therapy was not prescribed before and after surgery, unless it was necessary.

Postoperative follow-up evaluation included the following: clinical history, physical examination, 3-day bladder diary, upright cough stress test with bladder filling > 400 ml (measured by ultrasound), and subjective satisfaction assessment.

Objective cure was defined as the absence of urine leakage during the stress test, while subjective outcomes were assessed by means of the ICIQ-SF, the PGI-I scale [13], and a VAS with independent responses from 0 to 10 assessing the patient’s degree of satisfaction with continence (0 represents “not satisfied” and 10 “satisfied”) [14]. A combination of a PGI-I score ≤ 2 and a VAS score ≥ 8 was used to define the subjective success of the surgical procedure [9].

Follow-up visits were mandatory after 1, 10, and 17 years in all women, while intermediate visits were scheduled at the physician’s discretion.

The Declaration of Helsinki was followed, and preoperative written informed consent was obtained from all patients in this observational prospective evaluation. This study did not require ethical/institutional review board approval because normal clinical practice were followed [15].

## 3. Statistical Analysis

Statistical analysis was performed with IBM-SPSS v.17 for Windows (IBM Corp, Armonk, NY, USA). Continuous variables were reported as medians and interquartile ranges. We used the Chi-square (ꭓ^2^) test and ꭓ^2^ test for trends to analyze and compare the surgical outcomes during the follow-up. The Chi-square test can better assess if the successful effects of the surgical procedure tends to decrease over time, comparing the cure rates at different follow-up visits (1, 10, and 17 yr). The null hypothesis is that there is no association between the cure rate of TVT-O and time. Statistical significance was achieved when *p* < 0.05. One-way analysis of variance was used to compare the continuous series of variables in the comparison of the scores used to measure the subjective outcomes.

## 4. Results

A total of 70 patients who met the inclusion criteria underwent the TVT-O procedure. The baseline characteristics of the study group are reported in Table 1.

**Figure 1:**
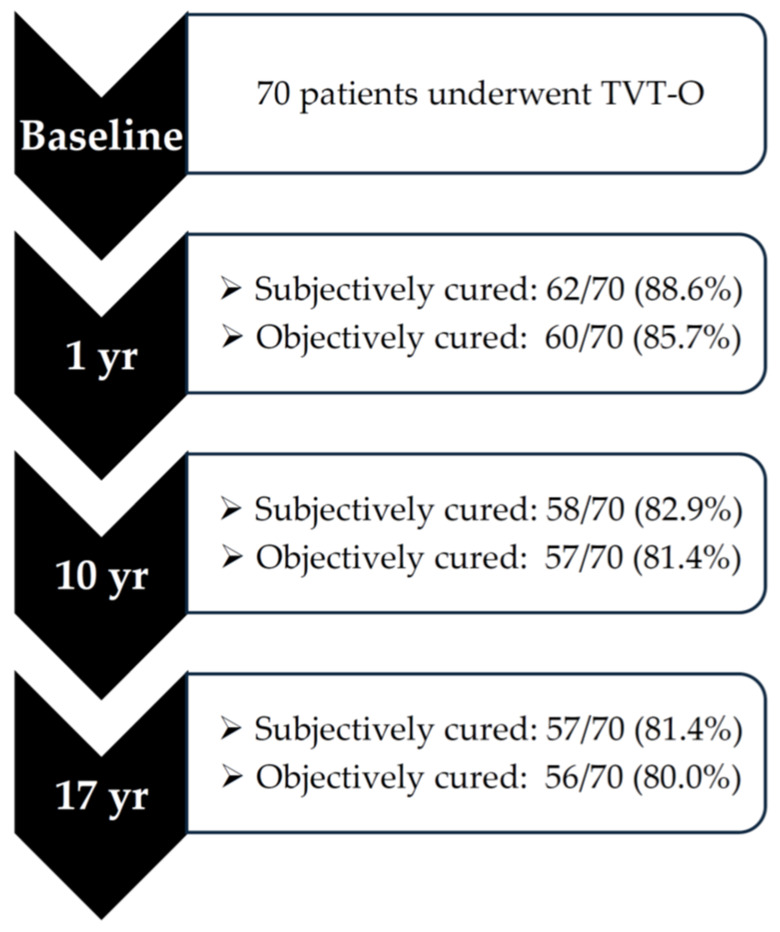
Progress of patients across this study.

During the study period, no patients were lost to follow-up, and all women completed the last evaluation at the 17-year mark. Data regarding the subjective and objective cure rates are summarized in Table 2 and Table 3. At the 17-year mark of follow-up, 57 out of 70 patients (81.4%) were subjectively cured (p for trend 0.24), and 56 out of 70 (80%) patients were objectively cured (p for trend 0.37), as shown in Figure 1.

These data do not reveal any significant variation in the surgical outcomes over the follow-up period. Only one patient required a second TVT-O surgical implantation at 10 years after the first procedure, while eight patients underwent a second treatment for SUI, specifically, urethral bulking agent (UBA) (Macroplastique^®^). Of these patients, four patients received UBA after 10 years of follow-up and another four patients after 17 years.

As reported in Table 3, long-term subjective outcome scores are not statistically influenced by time. In fact, 81% of women declared themselves as “very much improved” or “much improved”, and ICIQ-SF scores showed a significant reduction [17 (16–18) versus 0 (0–12)] at the last follow-up.

Table 4 reports the Clavien–Dindo classification of long-term complications of TVT-O.

We recorded seven (10%) complications of Clavien IIIa, corresponding to tape exposure (three occurred after 10 years and four after 17 years). Among these complications, one woman was symptomatic for dyspareunia and “hispareunia” [2]. Another two women in the study group complained of dyspareunia independent of exposure to the mesh. All patients with mesh exposure were treated with the partial removal and re-suture of the vagina, but only one developed the recurrence of SUI that required a second treatment with UBA. In all other cases, women reported a complete resolution of symptoms without any worsening of urinary continence. No significant bladder or urethral erosion was registered.

Three of seventy sexually active patients (15.7%) reported dyspareunia (without sling exposure) after 17 yr, when specifically asked whether they experienced pain during intercourse. In these cases, we prescribed local estrogenic therapy.

The onset of de novo OAB symptoms, classified as Clavien II, was reported by 13 of 70 women (18.5%) at the 17 yr follow-up. Eight of these women were classified as cases of dry OAB. At the last follow-up examination, only two patients reported persistent micturition dysfunction that required no treatment, while three patients complained of mild persistent pain (VAS score of 2/10 with no need of analgesic treatment). No significant bladder or urethral erosion was registered.

## 5. Discussion

This prospective cohort study reports the long-term outcomes of the TVT-O implantation for the treatment of female SUI. No study to date has ever reported on the results of transobturator MUS after 17 years of follow-up.

We demonstrated that TVT-O is an effective and safe procedure, reporting subjective and objective cure rates of 81.4% and 80%, respectively.

In addition, for the first time in the available literature, we identified the long-term risk of sling exposure. Although a low exposure rate was found, there was a significant upward trend over 17 years of follow-up. A possible explanation for this finding could be linked to the continuous reduction over time in the thickness of vaginal mucosa due to tissue atrophy in these patients during the 17 years of observation. Unfortunately, there are no studies specifically designed to evaluate whether the use of local estrogen supplementation or energy-based device treatments (laser or radiofrequency) can reduce the risk of long-term sling exposure. Despite this finding, no serious complications in terms of pain, dyspareunia, visceral perforation, or continence status were reported.

We also recorded an upward trend in de novo OAB. As reported in our previous study, in patients who are not clearly obstructed, it is difficult to explain the onset of de novo OAB. A plausible theory is that OAB could be caused by a weak urethral sphincter mechanism, resulting in funneling of the proximal urethra. It was postulated that when urine enters the proximal urethra, it produces sensory stimulation resulting in reflex bladder contraction since urethral afferent nerve activity can induce involuntary detrusor contraction [16].

Until a few years ago, the use of MUS for SUI treatment was considered the gold standard; however, after the US Food and Drug Administration (FDA) in 2019 [17], their role has been called into question. Furthermore, NICE guidelines [2] ruled out the transobturator approach among the options for the treatment of SUI. The most important reason for this recommendation was the higher rate of mesh exposure compared to the retropubic approach based on an ESTER systematic review [18]. Nevertheless, in this review, it was not clear whether or not the terms “erosion”, “extrusion”, and “exposure” were used consistently across individual studies, and most of them had a short follow-up period (less than 2 years). The authors concluded that although MUS is generally better than other surgical procedures, long-term data are lacking.

In fact, as reported by Guillot-Tantay et al. in a systematic review on long-term safety of synthetic midurethral sling implantation for the treatment of SUI in adult women [19], there are few safety data beyond 5 years, as 22.7% of the articles reported a follow-up after 10 years and 2.3% after 15 years. Interestingly, the authors found only four studies that reported the overall reoperation rates after 10 years for transobturator tape (TOT) (range between 5% and 15%), and only four studies for tension-free vaginal tape (TVT) (range between 2% and 17%). In randomized control trials (RCTs), the overall reoperation rates after 10 years were 0% and 1% for TOTs (two studies), and no study was published for TVTs. On the other hand, the most frequent complications reported were erosions (from 0% to 7% at 5 years for TVTs and from 0% to 19% for TOTs). In light of these data, they concluded that there is an urgent need to improve the safety monitoring of MUSs because data beyond 5 yr are rare, heterogeneous, and of insufficient quality.

Tomaselli et al., in their systematic review and meta-analysis [20], evaluated the long-term outcomes of retropubic MUS (RP-MUS) procedures and the medium-term outcomes of transobturator MUS (TO-MUS) procedures. The authors included a total of 49 studies (11 RCTs and 38 non-randomized studies, including prospective, retrospective, and cohort studies, with a total of 6.406 patients). Studies with a follow-up of at least 36 months for TO-MUS and 60 months for RP-MUS were considered. They found that RP-MUS had comparable objective cure rates (OR 1.15, 95% CI 0.75–1.76) but higher subjective cure rates than TO-MUS (OR 1.76, 95% CI 1.08–2.86). Bladder injuries were more common (OR 7.01, 95% CI 2.94–17.90), and vaginal erosions were less frequent for RP-MUS (OR 0.24, 95% CI 0.07–0.84). Furthermore, vaginal injuries were more common with TOT than with TVT-O (OR 7.96, 95% CI 1.15–157.9). The authors concluded that the high efficacy of these procedures is supported by a high safety profile and a restricted number of complications, which are rarely severe. However, further RCTs, especially those comparing TVT-O and TOT with objective cure rates and longer follow-up periods, are needed.

Previously, in a multicenter prospective study [4], we assessed the efficacy and safety of TVT-O 13 years after implantation for the treatment of only female SUI. This study, that was the longest-term evaluation to date, demonstrated that TVT-O is a highly effective and safe option for female SUI. Furthermore, we found that there is a significantly higher risk of having sling exposure over ten years after the procedure [4], as reported in 157 (2.5%) cases [*p* = 0.05]; however, the incidence was very low.

Even in the large retrospective study by Gurol-Urganci et al., [3], there was an increase in the rate of MUS mesh removal over time: 1.4% after 1 year, 2.7% after 5 years, and 3.3% after 9 years. This last finding was similar to our previous evaluation, but the risk of transobutorator insertion was lower than the risk after retropubic implantation (2.7 versus 3.6%; hazard ratio: 0.72 [95% CI, 0.62–0.84]).

In this study, once again, we have shown that TVT-O is an effective and safe option for female SUI, even in the long term. On the other hand, attention should be paid to the increased rate of mesh exposure, especially in the second decade after insertion. In fact, we identified seven cases of sling exposure at the 17-year follow-up (10%). It is interesting to note that this percentage was not too different from the rate of exposure after sacrocolpopexy reported by Nygaard et al. in the CARE trial [21], who found, after 7 years, the mesh exposure rate was 10.5%. Yet, no one doubts the role of this surgery for the treatment of apical prolapse, albeit with a higher rate of mesh exposure in a shorter period of time.

Moreover, in our study, patients were mildly symptomatic, and none required total sling removal. However, it is important to consider mesh exposure data, especially when the procedure is performed in young patients.

Perhaps, in this case, it could be better to propose, or at least consider, as the first treatment, other options such as bulking agents, with an efficacy that has been demonstrated in the long term and even in the case of SUI recurrence after MUS [22,23,24,25]. One of the most debated topics currently is what could be the most suitable alternative to mid-urethral slings in the future for the surgical treatment of female SUI. Some authors and guidelines are proposing to renew the use of Burch colposuspension, in particular with a minimally invasive approach (laparoscopic or robotic). A recently published randomized control trial compared laparoscopic Burch colposuspension (LBC) and single-incision midurethral sling (SIMS), demonstrating that the two procedures are associated with similar objective and subjective outcomes and the same complication rate [26]

In 2021, another study with a prospective matched cohort design aimed to compare the long-term outcomes of Burch colposuspension with a retropubic midurethral sling [27]. Again, the study showed no differences in success, patient satisfaction, or complications between the Burch colposuspension and the retropubic midurethral sling. However, the risk of posterior compartment prolapse surgery after Burch colposuspension increased. The reoperation rates for incontinence were similar in the two groups. Therefore, Burch colposuspension may be an acceptable surgical treatment for SUI but does not appear to be able to reduce the complication rate compared to MUS.

On the contrary, to avoid complications related to midurethral slings (MUSs), the available data on the use of urethral bulking agents (UBA) appear to be very promising. Many studies have shown that UBAs, and, in particular, Macroplastique, are associated with a significantly lower intra- and postoperative complication rate than MUS, showing a similar subjective satisfaction rate [28,29]. Also, in terms of improving sexual function, these procedures could be effective, with similar results to those reported for MUS, but with a lower rate of de novo dyspareunia [30,31].

The strengths of this study are as follows: (a) extremely homogeneous study population, with strict exclusion and inclusion criteria, (b) use of validated tools for the assessment of subjective and objective outcomes, and (c) no patients lost to follow-up at either study center.

On the contrary, the limitations of this study may include the following: (a) limited sample size and (b) lack of data regarding quality of life (QoL) (but there is no validated QoL questionnaire in Italian).

## 6. Conclusions

The long-term evaluation of the TVT-O procedure has shown that it is a highly effective and safe option for the treatment of female SUI. The subjective and objective long-term results were very satisfactory. However, the risk of tape exposure increased significantly 17 years after implantation, although no serious complications were reported, and no patient required the total complete removal of the sling.

## Figures and Tables

**Table 1:** Baseline patients’ characteristics.

Characteristics	n = 70
Age, yr, median (IQR)	57 (49–73)
BMI, kg/m^2^, median (IQR)	26.3 (23–36)
Menopausal, no. (%)	51 (72.8)
HRT, no. (%)	11 (15.7)
Recurrent UTI, no. (%)	3 (4.3)
Previous vaginal deliveries, median (IQR)	2 (1–7)
Infant birth weight ≥ 4000 g, no. (%)	8 (11.4)
Operative delivery (vacuum/forceps), no. (%)	5 (7.1)
Smoking habits, no. (%)	10 (14.2)

IQR: interquartile range; BMI: body mass index; HRT: hormone replacement therapy; UTI: urinary tract infection.

**Table 2:** Cure rates at 1 yr,10 yr, and 17 yr follow-up visit.

	1 yr	10 yr	17 yr	*p* Value
**Subjectively cured** Satisfied (N)	88.6% (62/70)	82.9% (58/70)	81.4% (57/70)	0.47 ^a^ 0.24 ^b^
**Objectively cured** Objectively cured (at stress test)	85.7% (60/70)	81.4% (57/70)	80.0% (56/70)	0.65 ^a^ 0.37 ^b^
**de novo Overactive Bladder** Onset of OAB	10% (7/70)	14.2% (10/70)	18.5% (13/70)	0.35 ^a^ 0.15 ^b^

^a^ Chi-square test ^b^ Chi-square test for trends.

**Table 3:** Subjective outcomes scores over time after TVT-O.

	Baseline	1 yr	10 yr	17 yr	*p* Value
**ICIQ-sf**	18 (16–18)	0 (0–6)	0 (0–14)	0 (0–12)	<0.0001 *
**“Significantly better” or “much better” on PGI-I**		62/70 (88.6%)	58/70 (82.9%)	57/70 81.4%	

Data are expressed as absolute number (%) or median (interquartile range); * One-way analysis of variance (ANOVA).

**Table 4:** Clavien–Dindo classification of long-term complications.

Complication	n = 70	Action
**CLAVIEN I**		
Persistence of groin pain no (%) Persistence of voiding dysfunction	3/70 (4.3%) 2/70 (2.8%)	Analgetics Observation
**CLAVIEN II**		
De novo overactive bladder, no. (%)	13/70 (18.5%)	Antimuscarinics/ Beta-adrenergics
De novo dyspareunia, no. (%)	3/19 * (15.7%)	Local estrogenic therapy
**CLAVIEN IIIa**		
Tape exposure, no. (%)	7 (10%)	Partial removal and re-suture

Data are expressed as absolute number (%); * Patients sexually active at 17 yr.

## Data Availability

The original contributions presented in the study are included in the article, further inquiries can be directed to the corresponding author.
